# The Effectiveness of a Guided Internet-Based Tool for the Treatment of Depression and Anxiety in Pregnancy (MamaKits Online): Randomized Controlled Trial

**DOI:** 10.2196/15172

**Published:** 2020-03-23

**Authors:** Hanna M Heller, Adriaan W Hoogendoorn, Adriaan Honig, Birit F P Broekman, Annemieke van Straten

**Affiliations:** 1 Department of Psychiatry Amsterdam Universitair Medische Centra Vrije Universiteit Amsterdam Amsterdam Netherlands; 2 Amsterdam Public Health Research Institute Amsterdam Netherlands; 3 GGZ inGeest Amsterdam Netherlands; 4 Department of Psychiatry OLVG Hospital Amsterdam Netherlands; 5 Department of Clinical Psychology Vrije Universiteit Amsterdam Amsterdam Netherlands

**Keywords:** pregnancy, depression, anxiety, internet, pregnancy outcome, treatment

## Abstract

**Background:**

Pregnant women with symptoms of depression or anxiety often do not receive adequate treatment. In view of the high incidence of these symptoms in pregnancy and their impact on pregnancy outcomes, getting treatment is of the utmost importance. A guided internet self-help intervention may help to provide more women with appropriate treatment.

**Objective:**

This study aimed to examine the effectiveness of a guided internet intervention (MamaKits online) for pregnant women with moderate to severe symptoms of anxiety or depression. Assessments took place before randomization (T0), post intervention (T1), at 36 weeks of pregnancy (T2), and 6 weeks postpartum (T3). We also explored effects on perinatal child outcomes 6 weeks postpartum.

**Methods:**

This randomized controlled trial included pregnant women (<30 weeks) with depressive symptoms above threshold (ie, Center for Epidemiological Studies Depression scale [CES-D] >16) or anxiety above threshold (ie, Hospital Anxiety and Depression Scale-Anxiety subscale [HADS-A] >8) or both of them. Participants were recruited via general media and flyers in prenatal care waiting rooms or via obstetricians and midwives. After initial assessment, women were randomized to (1) MamaKits online in addition to treatment as usual or (2) treatment as usual (control condition). MamaKits online is a 5-week guided internet intervention based on problem solving treatment. Guidance was was provided by trained students pursuing a Master's in Psychology. Outcomes were based on a Web-based self-report. Women in the control condition were allowed to receive the intervention after the last assessment (6 weeks postpartum).

**Results:**

Of the 159 included women, 79 were randomized to MamaKits online, 47% (79/37) of whom completed the intervention. Both groups showed a substantial decrease in affective symptoms on the CES-D, HADS-A, and Edinburgh Postnatal Depression Scale over time. In the intervention group, affective symptoms decreased more than that in the control group, but between-group effect sizes were small to medium (Cohen *d* at T3=0.45, 0.21, and 0.23 for the 3 questionnaires, respectively) and statistically not significant. Negative perinatal child outcomes did not differ between the 2 groups (χ^2^_1_=0.1; *P*=.78). Completer analysis revealed no differences in outcome between the treatment completers and the control group. The trial was terminated early for reasons of futility based on the results of an interim analysis, which we performed because of inclusion problems.

**Conclusions:**

Our study did show a significant reduction in affective symptoms in both groups, but the differences in reduction of affective symptoms between the intervention and control groups were not significant. There were also no differences in perinatal child outcomes. Future research should examine for which women these interventions might be effective or if changes in the internet intervention might make the intervention more effective.

**Trial Registration:**

Netherlands Trial Register NL4162; https://tinyurl.com/sdckjek

## Introduction

### Background

Depression and anxiety are common problems in women in the perinatal period. Major depressive disorder and anxiety disorders affect 7% to 15% of women during pregnancy [1-4]. The prevalence of symptoms of depression and anxiety is even higher, as they occur in 18% to 20% of the pregnant women [1,5,6]. Depression and anxiety are both associated with poor pregnancy outcomes [7], postpartum depression [4,8,9], and negative influences on child development [10-14]. Hence, effective treatment of these disorders is of the utmost importance.

Psychotherapeutic interventions such as cognitive behavioral therapy and interpersonal therapy have proven effective in treating perinatal depression and anxiety [15-17]. However, the implementation of effective treatment interventions is often hampered by factors relating to the pregnancy, for example, nonrecognition of the symptoms because of overlapping symptomatology with pregnancy itself [18], or by feelings of stigmatization, lack of time, problems with transportation, or difficulties arranging childcare [11,19,20]. Some of these barriers may be overcome by providing guided internet-based self-help interventions. Indeed, Web-based interventions are easier to access, have no waiting lists, allow anonymity, and can be carried out whenever and wherever the patient wants [19,21,22]. Moreover, because the therapeutic input is smaller than that in regular face-to-face treatments, internet-based interventions are likely to be less costly and more scalable. This is especially advantageous for disorders that are characterized by a combination of high prevalence and a low treatment-seeking rate, which is the case for pregnant women with depressive and anxiety symptoms. Although internet-based interventions proved effective in the general population [23,24] as well as postpartum [25], recent studies of internet-based interventions during pregnancy [26-28] showed varying success. Outcomes may have been influenced by differences in the methodology, content, and duration of these internet-based therapies.

Previously, we developed an internet-based problem solving treatment (PST) consisting of five modules and support provided by a trained coach, which proved effective for depressed and anxious people in general [29,30]. PST is a generic treatment that is used for different kinds of psychiatric problems, such as depression [31] and anxiety [32]. The core assumption of PST is that affective symptoms are generated when people become overwhelmed by practical problems they face in their daily lives. In PST, participants make a list of all their worries and problems and learn structured ways to resolve those problems. This approach makes them feel less overwhelmed, which in turn alleviates their mood.

Although the effectiveness of face-to-face PST has been firmly established [31], there is no evidence yet whether online guided PST might be effective in reducing symptoms of depression and anxiety in pregnant women.

### Objectives

For this study, we adapted the Web-based guided PST to provide an effective, easily accessible intervention for above-threshold affective symptoms in pregnant women. We hypothesized that the intervention would be effective (1) in reducing depressive and anxiety symptoms post intervention during pregnancy, at the end of pregnancy, and at 6 weeks postpartum and (2) in improving perinatal child outcomes, such as preterm birth, growth restriction, and breastfeeding initiation.

## Methods

### Study Design

We performed a randomized controlled trial with an intervention condition (internet-based PST) and a control group (care-as-usual). For ethical reasons, the participants in the control condition were also offered access to the intervention, but only after the last follow-up (6 weeks postpartum). Both groups were allowed to use concurrent treatment (care-as-usual) as well. The use of additional care was monitored through self-report.

The study protocol, information brochure, and informed consent form were approved by the Medical Ethics Committee of the VU University Medical Center (registration number 2013.275) and registered with the Dutch Trial Registry (NL4162). The tenets of the Declaration of Helsinki were observed. An extensive description of the study protocol can be found elsewhere [33].

### Participants

All participants were self-referred. They were recruited through articles and advertisement in national newspapers and magazines and through social media, pregnancy websites, and websites of patient’s associations. Information flyers and posters were also distributed in maternity clinics and in clinics for primary care nationwide. Pregnant women with symptoms of depression and/or anxiety were advised to visit our study website, where they could find more information about the study and were given the opportunity to register online. After registration, they received an informed consent letter by post. After returning the signed informed consent form, they were invited to complete the first Web-based questionnaire.

### Inclusion and Exclusion Criteria

Women aged 18 years and older were eligible if they were pregnant for less than 30 weeks, showed symptoms of depression or anxiety or both, and had sufficient access to the internet. Symptoms of depression were measured with the initial Web-based questionnaire using the Center for Epidemiological Studies Depression scale (CES-D) [34], and symptoms of anxiety were assessed using the Hospital Anxiety and Depression Scale-Anxiety subscale (HADS-A) [35]. Women were eligible to participate if their score on the CES-D was at least 16 or the score on the HADS-A was 8 or more. Women with severe depressive or anxiety symptoms (CES-D ≥25 or HADS-A ≥12) were also allowed to participate. However, we advised them to contact their general practitioner as well to check if another treatment or additional treatment was needed. We did not exclude them because internet-based PST has also proven to be effective for severe depressive and anxiety symptoms [29,36,37]. However, women were excluded if they reported intentions to harm themselves or to attempt suicide (assessed by one question of the Web Screening Questionnaire) [38].

During the trial, participants in the intervention group were allowed to receive additional care-as-usual, such as psychiatric treatment including psychotherapy or psychopharmacological drugs. Any additional treatments were monitored through participants’ self-reports at every assessment.

### Randomization

Women who were included in the study were randomized in a 1:1 ratio to the intervention condition versus the control condition. An independent researcher created a computer-generated randomization scheme based on blocks of 10 and provided the next randomization outcome to one of the coaches. This procedure ensured allocation concealment. The research assistant informed all the participants on the randomization outcome by email. The participants of the intervention group also received the name of the website and details on where and how they could log-in to start the intervention.

### Intervention

An existing evidence-based internet version of PST [29] was used. We adapted this version for pregnant women by adding one session of psychoeducation on pregnancy and affective symptoms and adjusting all the existing case examples to pregnancy-related case examples. The adapted Web-based intervention for pregnant women was named MamaKits online. The course consists of 5 modules, and participants are advised to try to carry out one module each week. Each module consists of information, examples of other pregnant women with depressive or anxiety symptoms carrying out the intervention, and homework assignments.

The intervention consists of 3 steps: (1) participants describe what really matters to them, (2) participants write down all their current worries and problems, and (3) participants make a plan for the future, in which they describe how they will try to accomplish those things that matter most to them. After that, they categorize the problems into three types: unimportant problems (problems unrelated to the things that matter to them), problems that can potentially be solved, and problems that cannot be solved (eg, the loss of a loved one). The core of the intervention consists of a structured approach to solve the potentially solvable problems. This approach consists of 6 steps: (1) write down a clear definition of the problem, (2) generate multiple solutions to the problem, (3) select the best solution, (4) work out a systematic plan for this solution, (5) carry out the solution, and (6) evaluate whether the solution has resolved the problem.

After each module, trained coaches (students pursuing Master’s in Psychology) provided feedback on the assignments via secured email. All coaches were trained for 4 hours in PST and providing feedback via secured email. They were trained by an experienced psychotherapist, who also provided the coaches with regular supervision. On average, the coaches gave 20 min of feedback per patient per module. The feedback was directed to helping the patient work through the intervention; the coaches answered questions if something was not clear and provided feedback on homework assignments. If a participant was delayed in submitting the homework, the coach sent a reminder by email, with a maximum of three emails and one phone call after that.

### Measures

#### Assessments

Assessments took place at baseline (T0), 10 weeks after baseline (T1), 4 weeks before the expected date of delivery (T2), and 6 weeks postpartum (T3). Participants who started the intervention after 24 weeks’ gestation were not assessed at T2, as the period between T1 and T2 would have been too short to expect any effects. All assessments were based on self-report and took place online. At baseline, we additionally collected demographic data and data on current mental treatment, parity, pregnancy duration, and previous and current pregnancy complications. At T1, we collected additional data about treatment satisfaction, and at T3, we additionally collected data on perinatal child outcomes.

#### Primary Outcomes

Primary outcomes were reduction in symptoms of depression and anxiety and perinatal child outcomes. Depression was measured with the Dutch version of the CES-D [34]. This scale has 20 self-rated items, each of which is scored from 0 to 3. The total score range is 0 (no depressive symptoms) to 60 (high number of depressive symptoms). The validity of the CES-D has been tested in different populations, including pregnant women [39,40] and also online [41]. Scores of 16 and higher represent a clinically significant level of depressive symptoms with a sensitivity of 0.82 to 1.00 and a specificity of 0.69 to 0.88 [37,38].

Anxiety was measured with the Dutch version of the HADS-A [35]. The HADS-A is a 7-item anxiety subscale of the HADS with item responses on a 0 to 3 scale. Total score range is 0 to 21. Higher scores indicate more anxiety. The questionnaire has been found to be reliable in the internet version [42]. The HADS-A has an optimal cut-off ≥8 with a sensitivity of 0.89 and a specificity of 0.75 [43].

Perinatal child outcomes were assessed through self-report and analyzed by calculating the differences between the percentages of women in the intervention and control condition who delivered preterm (gestational age <37 weeks), whose babies had a low birth weight for gestational age (weight ≤tenth percentile, according to the guidelines by the Dutch Association of Gynecologists and Obstetricians, based on data of the Dutch National Birth Register), who delivered with an emergency cesarean section or vacuum extraction, or who did not continue breastfeeding until 6 weeks postpartum.

#### Secondary Outcomes

Secondary outcomes were reduction in symptoms of depression as measured with the Edinburgh Postnatal Depression Scale (EPDS), additional psychological health care use, and treatment satisfaction. The EPDS [44] is a 10-item depression scale developed for women primarily in the postpartum period, but also in pregnancy. Depending on the trimester, the cut-off score varied worldwide from 6.5 to 14.5, and in the Netherlands, it varied from 10 to 11 [45,46]. Item response varies from 0 to 3, and the total score range is 0 to 30 [45]. Information about additional mental health care was obtained using the Trimbos/institute for Medical Technology Assessment, Erasmus University Rotterdam, questionnaire for Costs associated with Psychiatric Illness [47].

We also used the Client Satisfaction Questionnaire (CSQ-8). The CSQ-8, a questionnaire with 8 items measured on a 4-point scale, has good psychometric properties in the Dutch population [48]. We added several questions about the intervention, the website, and the feedback of the coach. These questions could be answered through visual analog scales (VASs).

#### Sample Size Considerations

The between-group effect size (Cohen *d*) at post test (T1) was assumed to be at least 0.40, as was demonstrated in previous studies using the same internet-based PST [29,30]. Using an alpha of .05 (2-tailed), a statistical power (1-beta) of 0.80, and an attrition rate of 30% (as seen in other internet-based therapies in depressed patients) [30], we calculated that we needed to enroll 143 respondents in each arm.

After reviewing the literature, we assumed that symptoms of major depressive disorder and any anxiety disorder affect 7% to 15% of women during pregnancy [1-4] and that about 17% of the pregnant women have mild affective symptoms in pregnancy [6]. On the basis of a yearly birth rate of 171,341 in the Netherlands [49], at least about 29,127 women would be eligible for screening. With an expected response rate of 1%, 291 women would be included. Therefore, inclusion was expected to be completed within 1 year.

#### Statistical Analysis

All data were analyzed according to intention-to-treat analysis (comprising all the participants who were randomized) as well as per-protocol analysis (focusing on the participants who completed the intervention, ie, a subset of the intention-to-treat sample).

Mean total scores (standard deviations) of the 3 questionnaires (CES-D, HADS-A, and EPDS) were computed for the intervention and control arms separately at different time points (T0, T1, T2, and T3). The internet-based PST intervention effect was tested with linear mixed model (LMM) analyses, while correcting for baseline differences in the depressive and anxiety symptoms. LMM analysis can handle missing data owing to dropout under the assumption that the data are missing-at-random. Adverse perinatal child outcomes were defined as having experienced any negative outcome and were also evaluated by means of chi-square tests. Statistical analyses were carried out with SPSS (version 24; IBM, Armonk, New York) and Stata (version 15; StataCorp, College Station, Texas) software.

## Results

### Inclusion, Study Flow, Study Termination, and Dropout

The inclusion period was extended from 1 to 3 years owing to a low inclusion rate (March 2014 until January 2017). After 3 years, we performed an interim analysis, which had not been planned in the study protocol, to decide if inclusion of additional participants (and applying for additional funding) would be worthwhile or not. We developed an interim analysis protocol, which was approved by the ethical board. We evaluated the intervention effect on the first primary outcome CES-D at posttest (T1) when 153 participants had been randomized. According to the interim analysis protocol, the trial would be stopped for efficacy if the estimated intervention effect (in terms of standardized mean difference) exceeded 0.54 (in other words, extra patients would not be needed because the power was enough to establish the effect with significance). Inclusion would also be stopped, for futility, if the intervention effect was below 0.29 (in other words, continuing with our previously planned number of patients would not be useful because even if this number was reached, the power would be insufficient to demonstrate the effect with significance). As the interim analysis provided an estimated effect size of 0.035, the inclusion of participants was terminated prematurely. Although the inclusion of new participants stopped, all measurements continued as scheduled for the participants already included.

**Figure 1:**
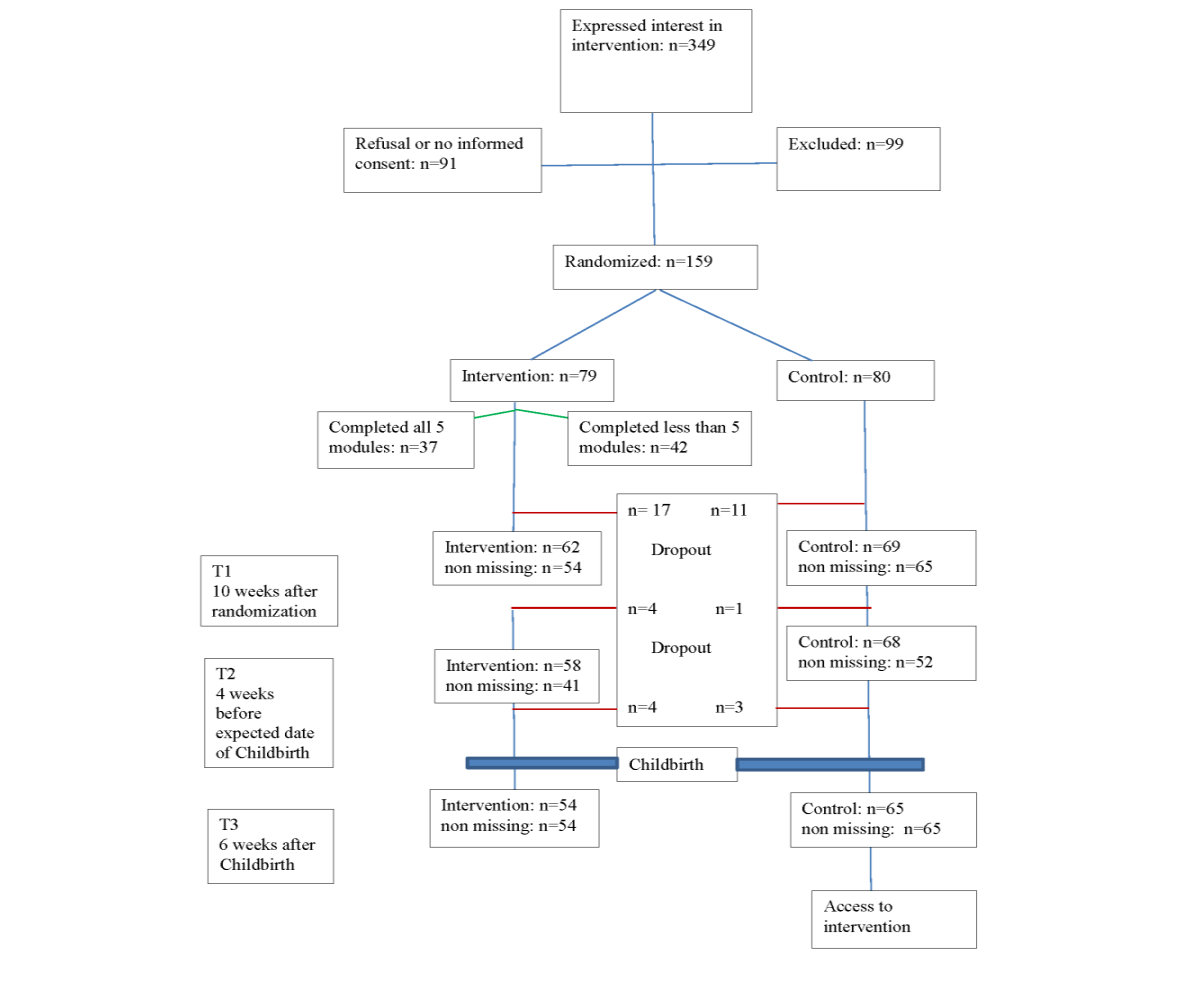
Flowchart of participants throughout the trial. Intervention: still in intervention group, but not everyone participated in this assessment; Control: still in control group, but not everyone participated in this assessment; Nonmissing: did participate in this assessment; Dropout: not in study anymore.

At the time of closure, a total of 349 women had expressed interest in the intervention. Of those women, 99 were excluded because they did not fulfill the inclusion criteria (eg, due to being pregnant beyond 30 weeks or due to not exceeding the required threshold for depression or anxiety scores). Of the remaining 250 women, 91 did not want to participate because of several reasons (eg, they already felt better or started another type of therapy). Of the originally planned 286 women, 159 were included in the study, as another 6 women were in the process of inclusion during the interim analysis. Of these 159 women, 79 were randomly allocated to the intervention group and 80 to the control group (Figure 1). Study dropout was 14% (11/80) in the control arm versus 22% (17/79) in the experimental arm at T1 (*P*=.20), 15% (12/80) versus 27% (21/79) at T2 (*P*=.07), and 19% (15/80) versus 32% (25/79) at T3 (*P*=.06). Overall, 60% (48/80) of the control group versus 43% (34/79) of the intervention group responded in all waves (T0, T1, T2, and T3) and 21% (17/80) of the control group versus 25% (20/79) of the intervention group missed either T1 or T2 (or both).

Of the 79 participants who were randomized to the intervention group, 37 (47%) completed all 5 modules of the intervention, 39 (49%) women completed at least four modules, 50 (63%) women completed at least three modules, 67 (72%) women completed at least two modules, 70 (89%) women completed at least one module, and 9 (11%) women did not even complete the first module. Reasons for nonadherence included being too busy (n=7), feeling better (n=4), need for other treatment (too sick; n=5), not being motivated (n=8), difficulties in confessing to the computer (n=1), intervention not meeting the expectations (n=3), and other reason/no reason given (n=14). There were no statistically significant differences between the baseline scores of treatment completers (having done all five modules) and noncompleters (having done less than five modules). The number of women using additional therapy was similar in both groups (*P*=.68, *P*=.82, and *P*=.73 at T0, T1, and T3, respectively).

### Description of Participants

In total, 159 women were randomized. Differences in baseline demographics between the internet-based PST group and the control group were small (Table 1). Most women were of native Dutch origin (134/159, 84.2%), highly educated (120/159, 75.4%), and employed (111/159, 69.8%). Differences between the intervention and the control group with respect to baseline severity scores of depression and anxiety (primary and secondary outcomes) were also small and nonsignificant.

**Table 1:** Sociodemographic and clinical characteristics at baseline for the intervention group and the control group (primary and secondary outcomes).

Demographic factors			Intervention, n=79	Control, n=80
Age (years), mean (SD)			32.08 (4.61)	31.94 (4.83)
**Background, n (%)**				
	Dutch		72 (91)	62 (78)
	Other		7 (9)	18 (23)
**Educationª, n (%)**				
	Low		4 (5)	0 (0.0)
	Middle		14 (18)	21 (26)
	High		61 (77)	59 (74)
**Marital status, n (%)**				
	In a relationship		76 (96)	76 (95)
	Living together		71 (90)	73 (91)
Employed, n (%)			57 (72)	54 (68)
**Pregnancy, n (%)**				
	**Duration by study entrance**			
		<12 weeks	5 (6)	11 (14)
		>12 and <26 weeks	48 (61)	44 (55.0)
		> 26 weeks	26 (33)	25 (31)
Nulliparous			42 (53)	36 (45)
Complications in previous pregnancy^b^			29 (60)	39 (72)
Complications in this pregnancy			9 (11)	7 (9)
**Previous mental health^c^, n (%)**				
	Depressive disorder		24 (30)	29 (36)
	Anxiety disorder		20 (25)	25 (31)
	Other mental problems		9 (11)	2 (3)
	No diagnosis		31 (39)	30 (38)
**Current treatment, n (%)**				
	Psychological treatment		31 (39)	34 (43)
	Psychotropic medication		12 (15)	14 (18)
**Affective symptoms, mean (SD)**				
	**Primary outcomes**			
		Center for Epidemiological Studies Depression	28.84 (7.54)	27.94 (9.04)
		Hospital Anxiety and Depression Scale-Anxiety	11.44 (3.50)	11.89 (3.38)
	**Secondary outcome**			
		Edinburgh Postnatal Depression Scale	14.27 (4.91)	13.96 (4.94)

^a^Dutch Standard Classification of Education: 2006–Edition 2016/’17, CBS, Statistics Netherlands.

^b^First pregnancies excluded.

^c^Note that women can be both in the category “depressive disorder” and in the category “anxiety disorder.”

### Effects on Mood Within the Intention-to-Treat Sample

**Figure 2:**
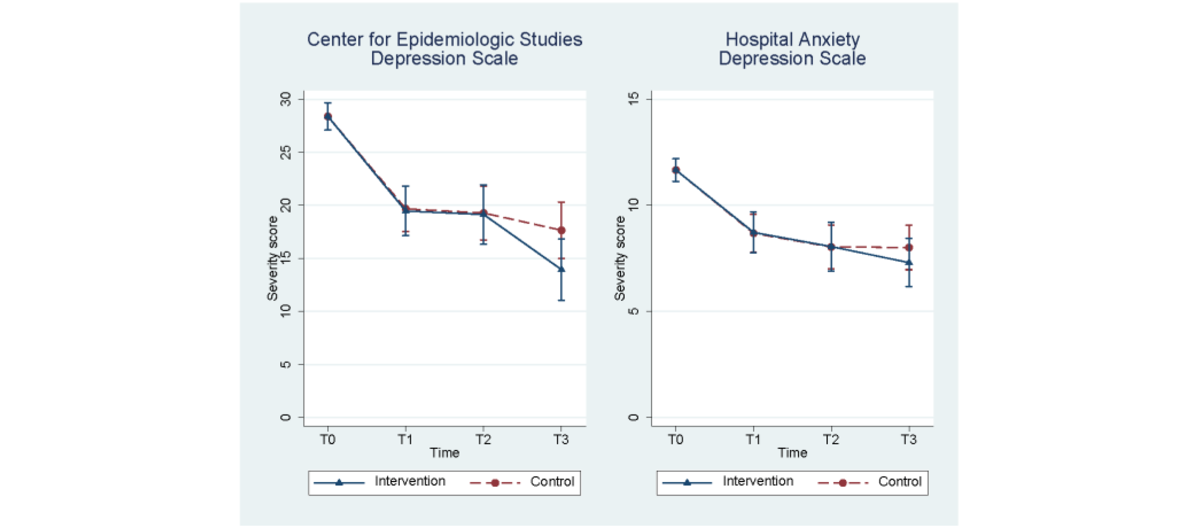
Predicted Center for Epidemiological Studies Depression and Hospital Anxiety and Depression Scale severity scores (primary outcomes) estimated using linear mixed models correcting for baseline differences. Measurements taken at T0: baseline; T1: 10 weeks after baseline; T2: 4 weeks before the expected date of delivery; T3: 6 weeks postpartum.

In the intervention group, large within-group effect sizes in primary and secondary outcomes were found between T0 and T1, T2 and T3 (Table 2). However, within-group effect sizes in the control group were also large (Figure 2). Differences in effects, as measured in the between-group effect sizes, were small and statistically insignificant (Table 3). The only exception to this finding was the medium effect size of the CES-D outcome on T3, but this was not significant either (*d*=0.45; *P*=.06).

**Table 2:** Mean scores (standard deviations) for affective symptoms (primary and secondary outcomes) considering the intervention group and the control group at baseline, 10 weeks after randomization, 4 weeks before expected birth date, and 6 weeks after child birth.

Condition			Baseline				10 weeks after randomization					4 weeks before expected birth date					6 weeks after child birth				
			n		Mean (SD)			n		Mean (SD)			n		Mean (SD)			n		Mean (SD)	
**Primary outcomes**																					
	**Center for Epidemiological Studies Depression**																				
		Intervention		79		28.8 (7.5)			54		19.5 (10.2)			41		19.7 (11.1)			54		13.8 (10.3)
		Waitlist control		80		27.9 (9.0)			65		18.6 (9.4)			52		18.6 (10.0)			65		16.8 (11.9)
	**Hospital Anxiety and Depression Scale-Anxiety**																				
		Intervention		79		11.4 (3.5)			54		8.4 (4.2)			41		7.9 (4.4)			54		7.1 (4.4)
		Waitlist control		80		11.9 (3.4)			65		8.6 (3.7)			52		7.9 (4.1)			65		7.9 (4.5)
**Secondary outcomes**																					
	**Edinburgh Postnatal Depression Scale**																				
		Intervention		79		14.3 (4.9)			54		9.5 (5.6)			41		9.0 (5.5)			54		8.0 (5.2)
		Waitlist control		80		14.0 (4.9)			65		8.9 (5.5)			52		8.2 (5.2)			65		8.7 (5.9)

**Table 3:** Estimated effects (unstandardized), test results, and effect sizes of the differences in primary and secondary outcomes (Center for Epidemiological Studies Depression, Hospital Anxiety and Depression Scale-Anxiety, and Edinburgh Postnatal Depression Scale) within groups and between the intervention group and the control group, using linear mixed model analysis at baseline, 10 weeks after randomization, 4 weeks before expected birth date, and 6 weeks after child birth after correction for scores at baseline.

Condition^a^			Estimated effect^b^	Test results			Effect size^c^ (Cohen *d*)		
				Test statistic, *z*	*P* value	Within control condition		Within intervention condition	Between groups
**Primary outcomes**									
	**Center for Epidemiological Studies Depression**								
		Intervention×T1^d^	−0.21	−0.14	.89	−1.05		−1.07	−0.03
		Intervention×T2^e^	−0.14	−0.08	.94	−1.10		−1.11	−0.02
		Intervention×T3^f^	−3.71	−1.87	.06	−1.29		−1.74	−0.45
	**Hospital Anxiety and Depression Scale-Anxiety**								
		Intervention×T1	0.04	0.07	.95	−0.87		−0.86	0.01
		Intervention×T2	0.02	0.02	.98	−1.06		−1.05	0.01
		Intervention×T3	−0.71	−0.92	.36	−1.06		−1.27	−0.21
**Secondary outcomes**									
	**Edinburgh Postnatal Depression Scale**								
		Intervention×T1	0.00	0.01	.10	−0.91		−0.91	0.00
		Intervention×T2	0.10	0.11	.91	−1.09		−1.07	0.02
		Intervention×T3	−1.12	−1.13	.26	−1.02		−1.25	−0.23

^a^The test on all three parameters tests the null hypothesis that all three intervention-by-timepoint interaction terms are zero, meaning that the course of the outcome variable within the intervention group is identical to the course of the outcome variable within the waitlist control group.

^b^Estimated effects (unstandardized) are the parameter estimates of the intervention-by-timepoint interaction terms and reflect the additional increase (or decrease) within the intervention group compared with the increase (or decrease) in the waitlist control group.

^c^Effect sizes (Cohen *d*) are standardized effects, obtained by dividing the unstandardized estimated effects by the standard deviation of the primary outcomes.

^d^T1: 10 weeks after randomization.

^e^T2: 4 weeks before expected birth date.

^f^T3: 6 weeks after child birth.

### Effects on Perinatal Child Outcomes Within the Intention-to-Treat Sample

The analyses of perinatal child outcomes revealed that 50.4% (60/119) of the women experienced one or more negative perinatal child outcomes or early cessation of breastfeeding. There was no statistically significant difference in these perinatal outcomes between the intervention group and the control group (Table 4).

**Table 4:** Perinatal child outcomes in the intervention group compared with the control group.

Perinatal child outcomes	Intervention, n (%)	Control, n (%)	*P* value
Preterm birth	4 (7)	1 (2)	.12
Small for gestational age	1 (2)	4 (6)	.24
Emergency cesarean section	7 (13)	9 (14)	.89
Vacuum extraction	7 (13)	9 (14)	.89
No breastfeeding initiation	9 (17)	5 (8)	.13
Stopped breastfeeding early	9 (17)	11 (17)	.31
At least one negative perinatal child outcome (including no or early cessation of breastfeeding)	28 (52)	32 (49)	.78

### Per-Protocol Analysis of Treatment Completers (Mood and Perinatal Outcomes)

Of all 79 intervention patients, 37 (47%) completed the whole intervention. We examined the effects for those patients compared with the controls and found no significant differences in any of the outcome measures. LMM analyses revealed predominantly small nonsignificant differences between group effect sizes, with the exception of the CES-D on T3, which had a significant, medium to high effect size (CES-D T1: *d*=−0.25 and *P*=.21, CES-D T3: *d*=−0.53 and *P*=.04, HADS T1: *d*=−0.04 and *P*=.85, HADS T3: *d*=−0.41 and *P*=.09, EPDS T1: *d*=−0.09 and *P*=.66, and EPDS T3: *d*=−0.27 and *P*=.25).

### Client Satisfaction

The CSQ-8 was completed at T1 by 53 intervention participants. The majority of the participants 87% (46/53) were satisfied with the help they received and 74% (39/53) would recommend the intervention to others. The total intervention was rated 7.1 (SD 1.6) on a 10-point VAS. The website was rated as fairly good to excellent by 83% (44/53) of the participants, and the feedback of coaches was also rated as fairly good to excellent by 83% (44/53) of the participants.

### Additional Psychological Health Care

Both groups used additional psychological health care interventions, and in all cases, these interventions consisted of outpatient care. There were no statistically significant differences between the groups in the use of additional psychological health care. This was 42% (25/54) in the intervention group and 46% (27/65) in the control group (*P*=.60).

## Discussion

### Principal Findings

To the best of our knowledge, this randomized controlled trial is the first to investigate the effects of offering Web-based guided PST to pregnant women with symptoms of depression and anxiety, with the purpose of reducing barriers for effective therapy. In both the intervention group and the control group, symptoms decreased significantly over time, till 6 weeks postpartum. Although this difference was more pronounced in the intervention group than in the control group, the between-group differences were small and not statistically significant. The only statistically significant difference was shown in the per-protocol analysis at T3 on depression*.* We consider this result a *lucky finding*, and therefore, we do not think that this result is clinically meaningful. Except for this outlier, the outcomes of the questionnaires did not differ much. The differences in outcomes on the CES-D were larger than those on the EPDS. This might be explained by the fact that the EPDS also contains anxiety items (question 4 and 5), assuming that the intervention had a smaller effect on anxiety than on depression. There were also no differences between the groups in perinatal child outcomes. Attrition was high, with 47% (37/79) women completing the whole intervention and 63% (50/79) women completing more than three modules. In both groups, many women (52/119, 43.6%) used additional psychological treatment.

Our results are not in line with those of other studies on the effects of face-to-face PST in pregnant women, or with those of studies on the effects of internet-based PST in general, or with those of other Web-based therapies for pregnant women. Studies on face-to-face PST delivered perinatally did show medium to high effect [16] on depression, and studies on internet PST among people recruited in the general population showed moderate effects on both depression and anxiety [29,30]. Of 2 other studies on Web-based cognitive behavioral treatment (CBT) for depression during pregnancy [26,50], one showed favorable effects on the follow-up of anxiety but not on depression [50], whereas the other showed a large effect on depression [26] and only a small nonsignificant effect on anxiety.

There might be several reasons why our findings are not in line with those of previous studies. One possible explanation could be the intervention itself. Although the pregnant women who were included were generally satisfied with the intervention, a considerable proportion of these women dropped out. This proportion was larger than that in the intervention of Loughnan [50]. Although we do not know the reasons for the high dropout rate, one reason might have been that the women had sufficiently recovered and did not need more therapy. However, the dropout rate might also indicate that the treatment was not optimal or not sufficiently adapted to the population. The participants might have preferred additional modules (eg, with psychoeducation about changing relationships and role transition) and more supplementary resources, or they might have preferred another type of treatment (eg, CBT), one more like the treatments offered in the above-mentioned studies [26,50]. Another possibility is that the women in our study might have preferred face-to-face therapy, which is the default treatment in the Netherlands. The other 2 trials on Web-based CBT for depression during pregnancy were performed in Australia and Sweden, where people might be more familiar with electronic health because of their inability to commute to health care facilities if they live in remote areas [26,50]. Nevertheless, the fact that almost half of all participants in this study did complete the whole intervention indicates that an internet treatment might be a useful addition to the existing mental health services in the Netherlands.

The second possible reason why our findings differ from those of previous studies is the difference in measuring techniques. In the study that found a significant treatment effect for depression, symptoms were measured with the Montgomery Åsberg Depression Rating Scale Self-report version [26]. This instrument might be more sensitive to picking up relevant changes, but as far as we know, it has not been validated in pregnancy, and the changes could also be related to the improvement of symptoms of pregnancy itself. Besides, both studies on Web-based CBT for depression during pregnancy also used the EPDS as secondary outcome, resulting in small nonsignificant treatment effects.

The third possible reason for the lack of effect in this study is the remarkable improvement in the control group. This suggests that the improvement in both groups might rather be explained by spontaneous recovery. In general, people seek treatment when they are feeling at their worst. It is not unusual that symptoms improve spontaneously afterward [51]. This improvement might also be explained by the use of additional psychological services. A considerable part of the intervention group as well as the control group used other psychological treatments, and the majority of them started treatment before the intervention and continued after starting the intervention.

The fourth possible reason is that the patients in our study were relatively healthy. They had less severe depressive symptoms than those in the studies on Web-based interventions that showed greater effects [26,27]. Studies with patients with more severe symptoms often demonstrate higher effects than studies with patients with less severe symptoms [37].

The fifth possible reason is that the intervention might have been offered at the wrong moment during pregnancy. Most participants (92/159, 57.8%) were in the second trimester of their pregnancy, and several systematic reviews concluded that interventions carried out toward the end of pregnancy or in the postpartum period might be more effective [16,52]. However, in view of the negative consequences of anxiety and depression in pregnancy, an early intervention is of the utmost importance. Although we did not offer our intervention later in pregnancy or in the postpartum period, as recommended, we did meet the other 2 mentioned requirements of a successful treatment, which are an individual approach and an approach targeted at an at-risk population [52].

### Strengths

Our study has several strengths. First, we created and tested the first internet version of evidence-based PST in a perinatal setting. Second, we had a relatively long follow-up of 20 weeks. Third, we used an array of different outcome measures, including perinatal child outcomes. Fourth, we allowed women of both groups to use concurrent treatment, including treatment as usual, which makes the results of our study compatible with clinical practice.

### Limitations

Despite all our efforts to increase the number of women included in the study (by seeking publicity and prolonging the study period by 2 years), the required number of participants was not obtained. Second, adherence to the intervention was limited. Third, perinatal child outcomes were self-reported, which makes them less objective. The fourth limitation is that there was a sampling bias of mostly native Dutch, employed, and highly educated women, which makes the results less representative of the general population. The fifth limitation is that because of trial reasons, and to keep the population more homogeneous, women in the last 10 weeks of pregnancy were excluded from starting the intervention because they might not be able to finish the treatment before delivery. This is a limitation because by setting this limit, we excluded a group of women who could have benefited from the intervention, and we also possibly reduced the inclusion rate. Furthermore, as we mentioned earlier, interventions carried out toward the end of pregnancy or in the postpartum period might have been more effective [16,52]. We, therefore, might have been able to demonstrate larger effects if the intervention had been offered during this period.

The sixth limitation is that due to the small inclusion sample, the prevalence of negative perinatal child outcomes is probably less reliable.

### Clinical Implications

The aim of our study was to improve the care for pregnant women with symptoms of depression or anxiety or both by offering a Web-based intervention with the intention to overcome perceived barriers to treatment. Although inclusion was low, attrition was high, and outcome differences between the intervention group and the control group were mostly nonsignificant, we still recommend investigating how adherence and the effectiveness might be improved by adjusting the Web-based intervention, as satisfaction with the offered modules was high and the intervention is easily applicable at low cost.

### Conclusions

To the best of our knowledge, this is the first study to examine a Web-based PST intervention in pregnant women. Although this study did not show a significant reduction in depression and anxiety in comparison with a control condition, Web-based interventions remain a practical, cost-effective, complementary, or alternative therapy modality for face-to-face treatment. Future research is needed to see if the intervention might be more successful if it is offered later in pregnancy or if it is better adapted to the pregnant population or both.

## Appendix

Multimedia Appendix 1 CONSORT-EHEALTH checklist. (V 1.6.1).

## Abbreviations

CBT: cognitive behavioral treatment
CES-D: Center for Epidemiological Studies Depression scale
CSQ-8: Client Satisfaction Questionnaire
EPDS: Edinburgh Postnatal Depression Scale
HADS-A: Hospital Anxiety and Depression Scale-Anxiety
PST: problem solving treatment
LMM: linear mixed model
VAS: visual analog scale
